# Underdetermined DOA Estimation of Quasi-Stationary Signals Using a Partly-Calibrated Array

**DOI:** 10.3390/s17040702

**Published:** 2017-03-28

**Authors:** Ben Wang, Wei Wang, Yujie Gu, Shujie Lei

**Affiliations:** 1 College of Automation, Harbin Engineering University, No. 145 Nantong Street, Harbin 150001, China; wangben@hrbeu.edu.cn (B.W.); leishujie8@163.com (S.L.); 2 Depaprtment of Electrical and Computer Engineering, Temple University, Philadelphia, PA 19122, USA; guyujie@hotmail.com

**Keywords:** DOA estimation, Khatri–Rao subspace, partly-calibrated array, quasi-stationary signal, underdetermined problem

## Abstract

Quasi-stationary signals have been widely found in practical applications, which have time-varying second-order statistics while staying static within local time frames. In this paper, we develop a robust direction-of-arrival (DOA) estimation algorithm for quasi-stationary signals based on the Khatri–Rao (KR) subspace approach. A partly-calibrated array is considered, in which some of the sensors have an inaccurate knowledge of the gain and phase. In detail, we first develop a closed-form solution to estimate the unknown sensor gains and phases. The array is then calibrated using the estimated sensor gains and phases which enables the improved DOA estimation. To reduce the computational complexity, we also proposed a reduced-dimensional method for DOA estimation. The exploitation of the KR subspace approach enables the proposed method to achieve a larger number of degrees-of-freedom, i.e., more sources than sensors can be estimated. The unique identification condition for the proposed method is also derived. Simulation results demonstrate the effectiveness of the proposed underdetermined DOA estimation algorithm for quasi-stationary signals.

## 1. Introduction

Direction-of-arrival (DOA) estimation is an important array signal processing technique which has found broad applications in radar, sonar, navigation and wireless communication [1]. In the past decades, a number of subspace-based DOA estimation algorithms have been developed including maximum likelihood (ML) [2,3,4], Capon beamforming [5,6,7], multiple signal classification (MUSIC) [8,9,10], and estimation of parameters via the rotational invariance technique (ESPRIT) [11,12]. Among them, MUSIC is the most popular one for passive localization. However, the recently developed time-reversal MUSIC algorithm can also implement active detection by utilizing the multistatic data matrix [13,14,15,16]. Moreover, [17] provides a learning-by-example approach based on the support vector machine (SVM), which exploits a multi-scaling procedure to enhance the angular resolution of the detection process in the region of incidence of the incoming waves. In addition, the sparse signal reconstruction-based DOA estimation algorithm [18] also shows good performance even with a single snapshot.

The methods mentioned above provide high accuracy DOA estimation when the array is well calibrated. However, their performances degrade when some of the array sensors are mis-calibrated. For instance, an array may suffer imperfect calibration [19] and/or mutual coupling [20] which may vary over time and environment, yielding perturbations in the sensor gain and phase characteristics. In this paper, we mainly focus on the calibration problem. Generally, the calibration methods are classified into three categories, i.e., pilot calibration, self-calibration and auto-calibration. The pilot calibration methods [21,22,23] utilize sources with known parameters, such as direction and location, to estimate the array uncertainties analytically by exploiting the mathematical model of the array response [24]. These kinds of algorithms have highly accurate estimation performance. However, they also highly depend on the accurate knowledge of the pilot sources, which may not always be available in practice. In addition, the pilot calibration may be expensive and time-consuming because of the off-line operation. The self-calibration methods [19,25] have aroused much interest because they do not require the pilot sources. The self-calibration methods can estimate the gain-phase uncertainties and DOA simultaneously, which makes them more attractive. The auto-calibration methods [24,26] do not use any external source for calibration. Instead, the array elements are transceivers transmitting to each other and these signals are used to remove the array uncertainties.

In practice, the response of some sensor elements is poorly known or even unknown when the array sensors are incompletely calibrated. This kind of array is known as partly-calibrated array [27,28,29]. A number of calibration algorithms have been proposed to ensure robust DOA estimation using partly-calibrated arrays [19,27,28,29]. Among them, an iterative method is proposed in [19] to perform array calibration and DOA estimation simultaneously. This approach, however, is suboptimal and works properly only when the array perturbations are small. An iterative algorithm is developed in [27] for DOA and sensor gain-phase joint estimation, where a good initialization is required to achieve the convergence to the global minimum. In [28], an ESPRIT-like algorithm is developed to achieve the joint estimation of DOA and unknown gains and phases without any iteration. In [29], a subspace-based DOA estimation approach is proposed, which is applicable to the partly-calibrated arrays composed of multiple well-calibrated subarrays of arbitrary known geometry. Moreover, the displacements of the sensor elements also lead to DOA estimation performance degradation. Utilizing the stochastic collocation method, a method is proposed in [30] to calculate the cumulative density functions (cdfs) of the DOA estimates due to a random displacement of one sensor element in an array. However, it does not achieve a robust DOA estimation result.

Limited by the degrees-of-freedom (DOFs), the number of sources is usually assumed to be less than the number of sensors, as in [27,28,29]. Hence, increasing the DOFs has attracted continuing interest. In the past few years, several algorithms have been developed to deal with the underdetermined DOA estimation problem, i.e., the number of sources is larger than the number of sensors, either by exploiting the high-order statistics [31] or the sparse array structures [32,33,34,35]. In [36], a Khatri–Rao (KR) subspace approach is proposed by exploiting the statistical properties of the quasi-stationary signals, which represent a class of commonly encountered nonstationary signals including speech and audio signals. The statistics of quasi-stationary signals are locally static over a short time period, but exhibit difference from one local time frame to another [36,37]. By exploiting such statistical properties, the KR subspace approach achieves 2(N−1) DOFs with an *N*-element uniform linear array (ULA).

In this paper, we propose a DOA estimation algorithm based on the KR subspace approach using the partly-calibrated array. The proposed method is a kind of self-calibration method. Due to the existence of the unknown sensor gain and phase perturbations, the conventional KR-MUSIC and KR-ESPRIT methods [36] generally suffer significant performance degradation. To address this issue, we develop a KR subspace-based robust DOA estimation algorithm by simultaneously estimating the unknown gain and phase uncertainties. By utilizing an ESPRIT-like algorithm, the DOA as well as the unknown gain and phase uncertainties are jointly estimated with closed-form expressions. As such, unlike other joint DOA estimation and calibration methods requiring iterative solution or spectral search, the proposed method can be effectively implemented to achieve a high accuracy. Different to the method in [28], the proposed method achieves higher DOFs by exploiting the KR subspace. After the array is fully calibrated with the estimated gain and phase errors, it enables the underdetermined DOA estimation problem to be solved with more sources than sensors. In order to reduce the computational complexity, we further propose a reduced-dimensional (RD) method by designing a RD matrix. Simulation results demonstrate the effectiveness of the proposed DOA estimation algorithm in the presence of several sensor gain and phase errors even with more sources than sensors.

The remainder of this paper is organized as follows. In Section 2, we first describe the signal model with a partly-calibrated array. Using the quasi-stationary signals, the KR subspace is then presented. In Section 3, we propose an underdetermined DOA estimation algorithm with a partly-calibrated array for quasi-stationary signals. Furthermore, a RD method is also provided. In Section 4, we discuss the condition for unique identification and computational complexity of the proposed method. Simulation results are given in Section 5 and Section 6 concludes this paper.

Notations used in this paper are as follows. Lower-case (upper-case) bold characters are used to denote vectors (matrices). ${\left(\cdot \right)}^{T}$, ${\left(\cdot \right)}^{*}$ and ${\left(\cdot \right)}^{H}$ denote the transpose, conjugate and Hermitian operations of a matrix or vector, respectively. E[·] stands for the expectation operator. ${∥\cdot ∥}_{F}$ denotes the Frobenius norm. trace[A] and rank[A] denote the trace and rank of matrix A, respectively. $C^{M\times N}$ denotes an M×N complex matrix or vector (when N=1). vec(x) is a vectorization operator that turns a matrix x into a vector by concatenating all columns. In addition, diag(x) denotes a diagonal matrix with the elements of x constituting the diagonal entries. $I_{N}$ denotes the N×N identity matrix, $1_{N}$ and $0_{N}$ stand for the N×1 all-one and all-zero vector, respectively. ⊗, ⊙ and ∘ denote the Kronecker product, Khatri–Rao product and Schur–Hadamart product, respectively. ∠(x) stands for the phase of variable *x*.

## 2. Signal Model

In this section, we first describe the partly-calibrated array signal model, then present the KR subspace utilizing the quasi-stationary signals.

### 2.1. Partly-Calibrated Array Model

Consider a partly-calibrated ULA with *N* omnidirectional antennas. We assume that there are *K* uncorrelated far-field sources impinging on the array with distinct DOAs $\theta ={\left[\theta_{1},\cdots ,\theta_{K}\right]}^{T}$. For the DOA estimation problem, it is a common scenario to use the first sensor as the reference. Thus, without loss of generality, it is practical to assume that the first $N_{c}$ sensors are well-calibrated, whereas the last $N-N_{c}$ sensors are uncalibrated with unknown, direction-independent gain and phase uncertainties [27,28]. It is worth noting that the algorithm remains effective when the calibrated sensors are located in arbitrary positions, provided that there is at least one pair of consecutive calibrated sensors [28]. The complex-valued baseband array received signal vector at time index *t* can be modeled as

(1) $$x\left(t\right)=\bar{A}s\left(t\right)+n\left(t\right),$$

where $\bar{A}=\Gamma \left(\gamma \right)A$ is the actual array manifold matrix, Γ(γ)=diag(γ) with $\gamma ={\left[1_{N_{c}}^{T},\rho_{1}e^{j\varphi_{1}},\cdots ,\rho_{N-N_{c}}e^{j\varphi_{N-N_{c}}}\right]}^{T}$ corresponding to the unknown sensor gain-phase vector. It is assumed that the sensor gains are nonzero, i.e., $\rho_{n}\neq 0,\forall n=1,\cdots ,N-N_{c}$. In addition, $A=[a\left(\theta_{1}\right),\cdots ,a\left(\theta_{K}\right)]$ is the assumed array manifold matrix with $a\left(\theta_{k}\right)={\left[1,e^{-j2\pi d/\lambda \sin\theta_{k}},\cdots ,e^{-j2\pi d/\lambda \left(N-1\right)\sin\theta_{k}}\right]}^{T}\in \;C^{N}$, where *d* and λ denote the inter-element spacing and the signal wavelength, respectively. $s\left(t\right)\;=\;{\left[s_{1}\left(t\right),\cdots ,s_{K}\left(t\right)\right]}^{T}\in C^{K}$ denotes the zero-mean quasi-stationary signal waveforms, and $n\left(t\right)∼\mathrm{CN}\left(0,\sigma_{n}^{2}I\right)\in C^{N}$ is the additive white Gaussian noise vector with zero-mean and covariance matrix $\sigma_{n}^{2}I_{N}$. We further assume that the source signals $s_{k}\left(t\right),k=1,\cdots ,K$ are independent of each other. In addition, the source signals and noise are also assumed to be uncorrelated.

### 2.2. Khatri–Rao Product Subspace

**Figure 1:**
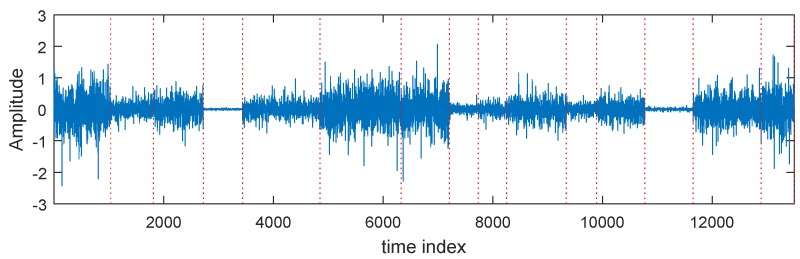
Quasi-stationary signals. The dashed lines are used to mark the local stationary intervals.

Quasi-stationary signals have a wide distribution in nature, such as speech and audio signals. Their second-order statistics remain static at a local time frame, however, they vary with different time frames. As shown in Figure 1, we illustrate the waveform of a quasi-stationary signal. Similar to [36], we model a quasi-stationary source signal $s_{k}\left(t\right)$ to have *M* non-overlapped time frames and the length of each frame is assumed to be *L*. In this case,

(2) $$E\left[{\left|s_{k}\left(t\right)\right|}^{2}\right]=\sigma_{mk}^{2},\forall t\in \left[\left(m-1\right)L,mL-1\right],m=1,\cdots M,$$

where $\sigma_{mk}^{2}$ is the signal power of $s_{k}\left(t\right)$ at the *m*th time frame. For the *m*th time frame, the sample covariance matrix is written as

(3) $$R_{m}=E\left[x\left(t\right)x^{H}\left(t\right)\right]=\bar{A}D_{m}{\bar{A}}^{H}+\sigma_{n}^{2}I_{N},$$

where $D_{m}=\Gamma \left([\sigma_{m1}^{2},\cdots ,\sigma_{mK}^{2}]\right)$. In practice, due to the finite number of snapshots, the covariance matrix is evaluated as ${\hat{R}}_{m}=\left(1/L\right)\sum_{t=(m-1)L}^{mL-1}x\left(t\right)x^{H}\left(t\right)$.

We vectorize ${\hat{R}}_{m}$ to an $N^{2}\times 1$ vector as:

(4) $$y_{m}=\mathrm{vec}\left({\hat{R}}_{m}\right)=\overset{˘}{A}d_{m}+\sigma_{n}^{2}i,$$

where $\overset{˘}{A}={\bar{A}}^{*}⊙\bar{A}$ is the generalized manifold matrix, $d_{m}={\left[\sigma_{m1}^{2},\cdots ,\sigma_{mK}^{2}\right]}^{T}$ and $i=\mathrm{vec}(I_{N})$. We can see that the expression of (4) is similar to the signal model in (1). To exploit the second-order properties of the quasi-stationary signals, we stack $y_{m}$ over all *M* time frames to yield the following matrix

(5) $$Y=\left[y_{1},\cdots ,y_{M}\right]=\overset{˘}{A}\Psi +\sigma_{n}^{2}i1_{M}^{T},$$

where $\Psi =[d_{1},\cdots ,d_{M}]$. Thus, the DOF of the array is extended.

As shown in [36], the noise covariance term $\sigma_{n}^{2}i1_{M}^{T}$ can be eliminated by utilizing the orthogonal complement matrix $P_{1_{M}}^{⊥}=I_{M}-\left(1/M\right)1_{M}1_{M}^{T}$, as

(6) $$\tilde{Y}=YP_{1_{M}}^{⊥}=\overset{˘}{A}\Psi P_{1_{M}}^{⊥},$$

the singular value decomposition (SVD) of which is expressed as

(7) $$\tilde{Y}=\left[E_{s}\;\;E_{n}\right]\left[\begin{array}{cc} \Sigma_{s} & 0\\ 0 & 0 \end{array}\right]\left[\begin{array}{c} V_{s}^{H}\\ V_{n}^{H} \end{array}\right],$$

where $E_{s}$ and $E_{n}$ are the left singular matrices corresponding to the nonzero and zero singular values, respectively, $V_{s}$ and $V_{n}$ are the respective right singular matrices, and $\Sigma_{s}$ is a diagonal matrix whose diagonal entries contain the nonzero singular values. Based on the subspace theory, matrix $E_{s}$ spans the same subspace as the generalized manifold matrix $\overset{˘}{A}$ which is denoted as the signal subspace. As such, these two matrices can be related by

(8) $$E_{s}=\overset{˘}{A}T,$$

where T is a K×K nonsingular matrix.

## 3. The Proposed Method

Due to the unknown sensor gain and phase uncertainties, the conventional KR subspace approach-based DOA estimation algorithms [36,37,38] suffer performance degradation or even failed operation. In this section, we first estimate the unknown gains and phases, then, the DOAs of the sources are estimated utilizing the ESPRIT-like algorithm based on the KR subspace approach.

### 3.1. Joint Parameters Estimation

The generalized manifold matrix $\overset{˘}{A}$ in (4) can be rewritten as

(9) $$\begin{array}{cc} \overset{˘}{A} & ={\left(\Gamma (\gamma )A\right)}^{*}⊙\left(\Gamma (\gamma )A\right)\\ & \;=\Gamma \left(\gamma^{*}⊗\gamma \right)\left(A^{*}⊙A\right)=\Gamma \left(\tilde{\gamma }\right)\tilde{A}, \end{array}$$

with $\tilde{\gamma }=\gamma^{*}⊗\gamma$ and $\tilde{A}=A^{*}⊙A$.

Similar to the conventional ESPRIT algorithm, the partly-calibrated ULA array is divided into two overlapping subarrays to exploit the rotational invariance property. Denote $\gamma_{1}$ and $\gamma_{2}$ as vectors containing the first and last N−1 elements of γ, and $A_{1}$ and $A_{2}$ matrices with the first and last N−1 rows of A, respectively. In addition, we define ${\tilde{\gamma }}_{1}=\gamma_{1}^{*}⊗\gamma$, ${\tilde{\gamma }}_{2}=\gamma_{2}^{*}⊗\gamma$, ${\tilde{A}}_{1}=A_{1}^{*}⊙A$ and ${\tilde{A}}_{2}=A_{2}^{*}⊙A$. By selecting the first and last N(N−1) rows of $E_{s}$, we have the two submatrices $E_{s1}$ and $E_{s2}$ as

(10) $$\begin{array}{c} E_{s1}=\Gamma \left({\tilde{\gamma }}_{1}\right){\tilde{A}}_{1}T,\\ E_{s2}=\Gamma \left({\tilde{\gamma }}_{2}\right){\tilde{A}}_{2}T. \end{array}$$

Using the rotational invariance property ${\tilde{A}}_{2}={\tilde{A}}_{1}\Phi$ with $\Phi =\Gamma \left({\left[e^{-j2\pi d/\lambda \sin\theta_{1}},\cdots ,e^{-j2\pi d/\lambda \sin\theta_{K}}\right]}^{T}\right)$, we can further obtain the following equation from the result of (10)

(11) $$\Gamma \left(Y\right)E_{s2}=E_{s1}\Pi ,$$

where $\Pi =T^{-1}\Phi T$, $\Gamma \left(Y\right)=\Gamma \left({\tilde{\gamma }}_{1}\right)\Gamma \left({\tilde{\gamma }}_{2}^{-1}\right)=\Gamma \left({\tilde{\gamma }}_{1}\circ {\tilde{\gamma }}_{2}^{-1}\right)$ with

(12) $$\begin{array}{cc} Y={\tilde{\gamma }}_{1}\circ {\tilde{\gamma }}_{2}^{-1} & =\left(\gamma_{1}^{*}⊗\gamma \right)\circ {\left(\gamma_{2}^{*}⊗\gamma \right)}^{-1}\\ & =\left(\gamma_{1}^{*}\circ {\left(\gamma_{2}^{*}\right)}^{-1}\right)⊗1_{N}. \end{array}$$

More specifically, Y can be expressed as

(13) $$\begin{array}{c} Y=[1_{\left(N_{c}-1\right)}^{T},{\left(\rho_{1}e^{j\varphi_{1}}\right)}^{-1},\rho_{1}e^{j\varphi_{1}}{\left(\rho_{2}e^{j\varphi_{2}}\right)}^{-1},\cdots ,\;\;\;\;\;\;\;\;\;\;\;\;\rho_{N-N_{c}-1}e^{j\varphi_{N-N_{c}-1}}{\left(\rho_{N-N_{c}}e^{j\varphi_{N-N_{c}}}\right)}^{-1}{]}^{H}⊗1_{N}. \end{array}$$

In the underlying problem with uncalibrated sensors, both Γ(Y) and Π in (11) are unknown. As such, the conventional ESPRIT algorithm [39] cannot be applied directly. Here, Γ(Y) and Π can be estimated by solving the following optimization problem

(14) $$\begin{array}{cc} \min_{Y,\Pi }\; & {∥\Gamma \left(Y\right)E_{s2}-E_{s1}\Pi ∥}_{F}^{2}\\ s.t.\; & WY=1_{(N_{c}-1)N}, \end{array}$$

where W is defined as

$$W=\left[\begin{array}{cc} I_{(N_{c}-1)N} & 0_{\left(N_{c}-1\right)N\times \left(N\left(N-1\right)-\left(N_{c}-1\right)N\right)} \end{array}\right].$$

The equality constraint in (14) is introduced to ensure that the first $N_{c}$ sensors take the calibrated gain and phase values. When the calibrated sensors are in arbitrary positions, the matrix W should be re-arranged to ensure that the corresponding sensors are well-calibrated.

With some simplifications as shown in the Appendix A, the original optimization problem (14) can be further reformulated as

(15) $$\begin{array}{cc} \min_{Y}\; & Y^{H}\left({\left(E_{s2}E_{s2}^{H}\right)}^{T}\circ P\right)Y\\ s.t.\; & WY=1_{(N_{c}-1)N}, \end{array}$$

which can be effectively solved using the Lagrange multipliers method. The matrix P is expressed as $P=I_{N(N-1)}-E_{s1}{\left(E_{s1}^{H}E_{s1}\right)}^{-1}E_{s1}^{H}$. The detailed derivation of the solution is given in Appendix B. The solution of the optimization problem (15) is expressed as

(16) $$\hat{Y}=Q^{-1}W^{T}{\left(WQ^{-1}W^{T}\right)}^{-1}1_{(N_{c}-1)N},$$

where $Q={\left(E_{s2}E_{s2}^{H}\right)}^{T}\circ P$. Note that Q is required to be nonsingular to estimate the $\hat{Y}$. In the case of infinite snapshots, Q is a nonsingular matrix. To ensure that Q is also a nonsingular matrix with finite snapshots in practice, diagonal loading [40,41] is a possible method to handle this problem. In addition, from the extensive experiments with finite snapshots that we have made, the matrix Q is always nonsingular. Thus, it is not necessary to use the diagonal loading method for Q in general [28].

Combining with (13), the *i*th entry of $\hat{\gamma }$ can be estimated as

(17) $${\hat{\gamma }}_{i}={\left(\prod_{n=N_{c}}^{i-1}T\left(n\right)\right)}^{-1},i=N_{c}+1,\cdots ,N,$$

where $T\left(n\right)=\frac{1}{N}\sum_{l=(n-1)N+1}^{nN}{\hat{Y}}_{l}^{*}$, and ${\hat{Y}}_{l}$ is the *l*th element of $\hat{Y}$.

By substituting the estimated $\hat{\overset{˘}{\gamma }}$ in (A8) into (A1), Π can be estimated as $\hat{\Pi }$. Since Π and Φ are similar matrices, the eigenvalues of Π are the diagonal entries of Φ and the columns of T are the eigenvectors of Π. Thus, the *k*th signal DOA can be estimated by

(18) $${\hat{\theta }}_{k}=\arcsin\left(-\frac{\lambda }{2\pi d}∠({\hat{\upsilon }}_{k})\right),k=1,\cdots ,K,$$

where ${\hat{\upsilon }}_{k}$ is the *k*th eigenvalue of $\hat{\Pi }$.

### 3.2. The Proposed RD Method

Once the unknown sensor gain-phase vector $\hat{\gamma }$ is estimated, the sensor array can be calibrated and the corresponding signal subspace can be expressed as

(19) $${\hat{E}}_{s}=\Gamma \left({\hat{\tilde{\gamma }}}^{-1}\right)E_{s}=\tilde{A}T,$$

where $\hat{\tilde{\gamma }}={\left({\hat{\gamma }}^{-1}\right)}^{*}⊗\hat{\gamma }$.

Owing to the fact that there only exist 2N−1 distinct rows in $\tilde{A}$, the effective dimension of $\tilde{A}$ can be reduced without any performance loss [36]. Let $\tilde{A}=GB$, where G is an $N^{2}\times \left(2N-1\right)$ RD matrix and $B=[b_{1},\cdots ,b_{K}]$ with $b_{k}=[e^{j2\pi \left(d/\lambda \right)\left(N-1\right)\sin\theta_{k}},\cdots ,e^{j2\pi \left(d/\lambda \right)\sin\theta_{k}},1,e^{-j2\pi \left(d/\lambda \right)\sin\theta_{k}},\cdots ,$ $e^{-j2\pi \left(d/\lambda \right)\left(N-1\right)\sin\theta_{k}}]\in C^{2N-1}$.

With ${\hat{E}}_{s}=\tilde{A}T$ and $\tilde{A}=GB$, the RD signal subspace can be expressed as

(20) $${\hat{E}}_{ds}=\Theta G^{T}{\hat{E}}_{s}=\Theta G^{T}\tilde{A}T=BT,$$

where $\Theta ={\left(G^{T}G\right)}^{-1}$.

To apply the ESPRIT algorithm, we construct two overlapping matrices by selecting the first 2N−2 and last 2N−2 rows of ${\hat{E}}_{ds}$, which are denoted as ${\hat{E}}_{ds1}$ and ${\hat{E}}_{ds2}$, respectively. We have

(21) $$\begin{array}{c} {\hat{E}}_{ds1}=B_{1}T\\ {\hat{E}}_{ds2}=B_{2}T, \end{array}$$

where $B_{1}$ and $B_{2}$ denote the first 2N−2 rows and last 2N−2 rows of B, respectively. Obviously, $B_{1}$ and $B_{2}$ hold the rotational invariance property, i.e., $B_{2}=B_{1}\Phi$. Thus, the DOAs can be estimated by utilizing the ESPRIT algorithm.

Although the proposed method is implemented using a ULA, the proposed method is applicable on other kinds of arrays which hold rotational invariance property, such as uniformly circle arrays (UCAs).

## 4. Condition for Unique Identification and Computational Complexity

First, we discuss the condition under which the true DOA can be uniquely estimated, which is equivalent to finding the condition for ${\hat{E}}_{ds1}$ and ${\hat{E}}_{ds2}$ to have the full column rank.

Since $\tilde{\gamma }$ is an $N^{2}\times 1$ vector with nonzero entries, we have $\mathrm{rank}\left[\Gamma (\tilde{\gamma })\right]=N^{2}$. Also note that $\overset{˘}{A}=\Gamma \left(\tilde{\gamma }\right)\tilde{A}=\Gamma \left(\tilde{\gamma }\right)GB$, rank[G]=2N−1 and rank[B]=min{2N−1,K}, the rank of $\overset{˘}{A}$ is given as

(22) $$\mathrm{rank}\left[\overset{˘}{A}\right]=\min\left\{\mathrm{rank}\left[\Gamma (\tilde{\gamma })\right],\mathrm{rank}\left[G\right],\mathrm{rank}\left[B\right]\right\}=\min\left\{N^{2},2N-1,K\right\}.$$

For a multi-sensor array, N>1, thus $N^{2}>2N-1$, and $\mathrm{rank}\left[\overset{˘}{A}\right]=\min\left\{2N-1,K\right\}$. Then, the full column rank condition for $\overset{˘}{A}$ is guaranteed as long as K≤2N−1 is satisfied.

Since the DOAs are estimated by exploiting the ESPRIT algorithm, the KR subspaces ${\tilde{E}}_{ds1}$ and ${\tilde{E}}_{ds2}$ will lose one DOF. As a result, the proposed method can resolve up to K≤2(N−1) sources. Compared with the standard ESPRIT method which only resolves up to N−1 sources, the proposed method is very attractive when there are more sources than sensors.

Next, we discuss the computational complexity of the proposed method. The SVD operation requires $O\{N^{2}M^{2}\}$. To estimate the unknown gain-phase error using (A8), $O\{N^{3}{\left(N-1\right)}^{2}\left(N_{c}-1\right)+N^{3}\left(N-1\right)\left(N_{c}-1\right)\left(N+N_{c}-2\right)+N^{3}\left(N_{c}-1\right)\}$ is required. In this paper, the ESPRIT algorithm is applied to perform DOA estimation, the main computational load is the eigenvalue decomposition (EVD) process which requires $O\{N^{3}{\left(N-1\right)}^{3}\}$. Utilizing the proposed RD method, the dimension of the signal subspace is reduced from N(N−1)×K to (2N−2)×K. Thus, the computational load for EVD operation becomes $O\{{\left(2N-2\right)}^{3}\}$. We summarize the computational complexities of the two methods in Table 1. It is clear that the computational complexity is significantly reduced by using the proposed RD method.

## 5. Simulation

In this section, simulation results are provided to demonstrate the effectiveness of the proposed DOA estimation algorithm based on the KR subspace approach using a partly-calibrated array.

In the first simulation, we consider a 10-element (N=10) partly-calibrated ULA with half-wavelength interelement spacing. The first five sensors are assumed to be well-calibrated, i.e., $N_{c}=5$, while the last five sensors are uncalibrated with direction-independent gain and phase uncertainties, $\gamma =[1_{5}^{T},0.8e^{j\pi /5},1.2e^{j\pi /5},1.53e^{-j\pi /5},0.75e^{j\pi /4},$ $1.36e^{-j\pi /3}{]}^{T}$. Assume that there exist four far-field sources with DOAs $-25^{\circ },10^{\circ },20^{\circ }$ and $30^{\circ }$, respectively. The frame period of each source is set as L=1024 and the number of frames is M=50. The signal-to-noise ratio (SNR) is defined as [36]

(23) $$\mathrm{SNR}=\frac{\frac{1}{T}\sum_{t=1}^{T}E\left[∥\bar{A}{s\left(t\right)∥}^{2}\right]}{E\left[{∥n\left(t\right)∥}^{2}\right]},$$

where T=LM. In the following simulation, we use 100 Monte Carlo trials to evaluate the performance of the proposed method.

In Table 2, we present the performance of the proposed gain-phase uncertainties estimation algorithm. The mean value and standard deviation results are calculated at a fixed SNR of 10 dB. It is evident that the proposed gain-phase estimation method achieves accurate gain and phase estimation.

**Figure 2:**
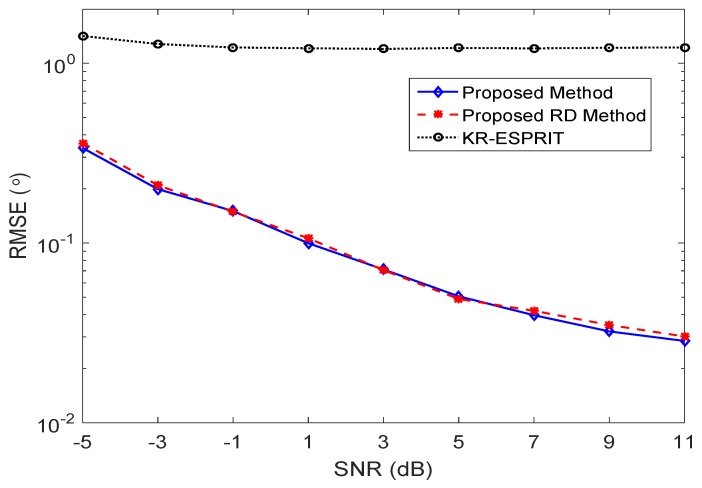
Root mean square error (RMSE) of DOA estimation versus SNR.

In Figure 2, the root mean square error (RMSE) of the proposed ESPRIT-like algorithm is compared with that of the KR-ESPRIT algorithm [36] at different SNRs. Here, the RMSE of DOA estimation is defined as

(24) $$\mathrm{RMSE}=\sqrt{E\left[\frac{1}{K}\sum_{k=1}^{K}{\left|{\hat{\theta }}_{k}-\theta_{k}\right|}^{2}\right]},$$

where ${\hat{\theta }}_{k}$ is the DOA estimation of the *k*th source. As shown in Figure 2, the KR-ESPRIT algorithm suffers significant performance degradation, while the proposed method shows accurate DOA estimation results. It also verifies that the proposed RD ESPRIT algorithm achieves almost the same DOA estimation without performance loss.

**Figure 3:**
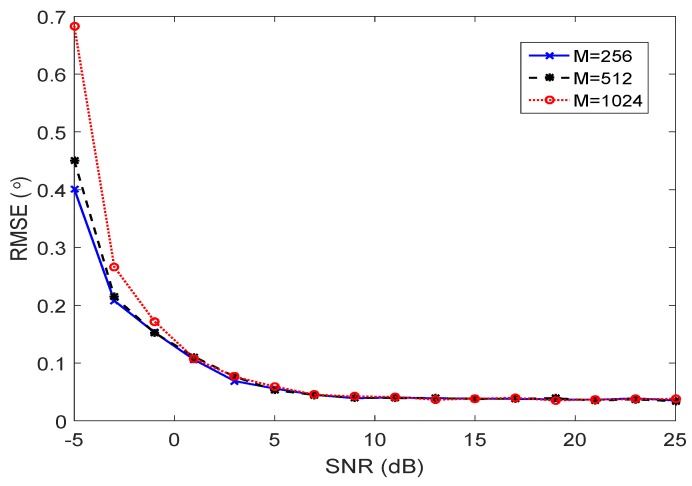
Estimation performance versus performance with different frame period lengths.

To evaluate the effect of frame length on estimation performance, we compare the RMSEs with different frame period lengths. In this simulation, the proposed RD method is used. Without loss of generality, the total number of snapshots used in this simulation is fixed as T=ML=51,200, and *M* varies with 256, 512 and 1024. From Figure 3, we can claim that with the increase of *M*, the performance degrades in the low SNR cases. In the high SNR cases, almost the same performance can be achieved with different frame periods. Note that the estimation of KR signal subspace $E_{s}$ requires a sufficient number of frames. When *L* becomes small, the estimation of $E_{s}$ will be distorted, which leads to a degraded estimation performance in the low SNR cases.

In the next simulation, we consider the underdetermined DOA estimation case. Consider a 5-element partly-calibrated ULA with $\gamma ={\left[1,1,1.2e^{j\pi /4},1,0.86e^{-j\pi /6}\right]}^{T}$. Assume that there exist six sources (K=6) with DOAs $-35^{\circ },-25^{\circ },-15^{\circ },0^{\circ },20^{\circ }$, and $30^{\circ }$, respectively. Here, the SNR is set as 10 dB. In addition, the frame length is selected as L=1024, and the total number of snapshots is set as T=51,200.

**Figure 4:**
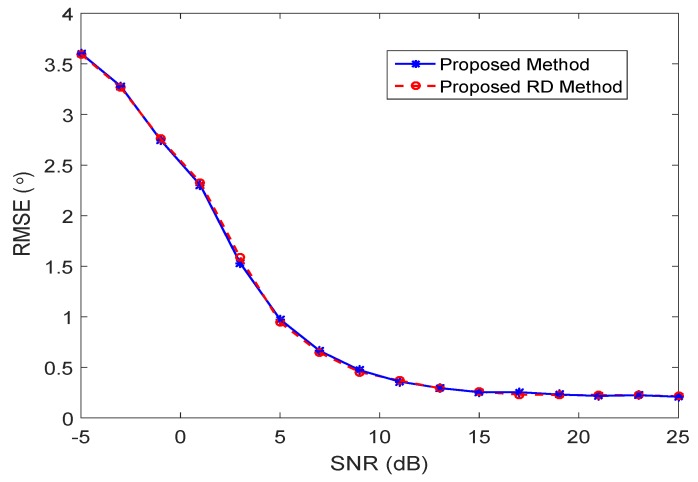
Performance comparison with the underdetermined case.

The performance comparison using the proposed algorithm method and the proposed RD method is plotted in Figure 4. It demonstrates that the proposed methods work well even when the number of sources is greater than the number of sensors. In addition, the proposed RD method achieves almost the same estimation performance with reduced complexity.

The gain and phase uncertainties estimation with the underdetermined case is presented in Table 3. The SNR used in this experiment is 10 dB. From Table 3, we can see that the gain-phase uncertainties estimation still has a solid performance.

**Figure 5:**
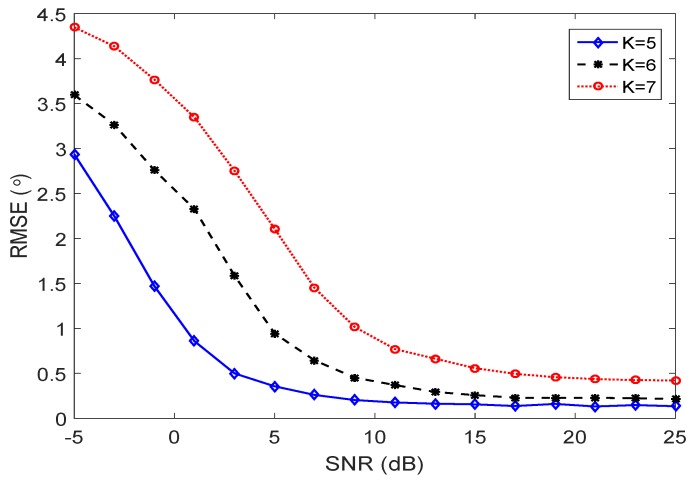
Estimation performance versus performance with different numbers of sources using the proposed RD method.

The performance of the proposed DOA estimation method with the underdetermined case is evaluated by calculating the RMSEs versus SNRs with a different number of targets. As is shown in Figure 5, with the increased number of sources, the performance is heavily degraded in the low SNR case. In the high SNR case, the RMSE curves converge and the proposed method achieves good performance.

## 6. Conclusions

In this paper, we proposed a DOA estimation algorithm for quasi-stationary signals based on the KR subspace approach with a partly-calibrated array. We first developed a closed-form expression to estimate the unknown sensor gains and phases in the KR subspace. Then, the signal DOAs were estimated by applying the ESPRIT algorithm. In order to reduce the computational complexity, a RD method was developed. Finally, we derived the unique identification condition for the proposed method and the DOFs of the proposed method were also analyzed. We have analytically shown and verified through simulations that the proposed RD method can estimate the DOAs of more sources than sensors with a partly-calibrated array. In the future, it will be interesting and practical to study the correlated/coherent sources scenario based on the KR subspace method with a partly-calibrated array.

## Figures and Tables

**Table 1:** Comparison of the computational complexity of two methods.

Method	SVD Operation	Gain-Phase Error Estimation	EVD for DOA Estimation
Proposed method	$O\left\{N^{2}M^{2}\right\}$	$O\left\{N^{3}{\left(N-1\right)}^{2}\left(N_{c}-1\right)+N^{3}\left(N-1\right)\left(N_{c}-1\right)\left(N+N_{c}-2\right)+N^{3}\left(N_{c}-1\right)\right\}$	$O\left\{N^{3}{\left(N-1\right)}^{3}\right\}$
Proposed RD method			$O\left\{{\left(2N-2\right)}^{3}\right\}$

**Table 2:** Performance of gain and phase estimation.

(a) Gain Estimation Results				(b) Phase Estimation Results (Radian)			
Index	True Value	Mean	STD	Index	True Value	Mean	STD
${\hat{\rho }}_{1}$	0.8000	0.8056	0.0049	${\hat{\varphi }}_{1}$	0.6283	0.6286	0.0055
${\hat{\rho }}_{2}$	1.2500	1.2510	0.0085	${\hat{\varphi }}_{2}$	−1.0472	−1.0473	0.0061
${\hat{\rho }}_{3}$	1.5300	1.5344	0.0118	${\hat{\varphi }}_{3}$	−0.6283	−0.6284	0.0074
${\hat{\rho }}_{4}$	0.7500	0.7608	0.0077	${\hat{\varphi }}_{4}$	0.7854	0.7855	0.0092
${\hat{\rho }}_{5}$	1.3600	1.3700	0.0164	${\hat{\varphi }}_{5}$	−1.0472	−1.0481	0.0099

**Table 3:** Performance of gain and phase estimation with the underdetermined case.

(a) Gain Estimation Results				(b) Phase Estimation Results (Radian)			
Index	True Value	Mean	STD	Index	True Value	Mean	STD
${\hat{\rho }}_{1}$	1.2000	1.1990	0.0138	${\hat{\varphi }}_{1}$	0.7853	0.7836	0.0093
${\hat{\rho }}_{2}$	0.8600	0.8749	0.0267	${\hat{\varphi }}_{2}$	−0.5236	−0.5269	0.0123

## Appendix A

In this Appendix, we provide the detailed derivation for the optimization problem (15).

As shown in (11), Π can be calculated based on the least squares method as

(A1) $$\Pi ={\left(E_{s1}^{H}E_{s1}\right)}^{-1}E_{s1}^{H}\Gamma \left(Y\right)E_{s2}.$$

Substituting (A1) back to (14) results in

(A2) $$\begin{array}{cc} \min_{Y}\; & ∥P\Gamma \left(Y\right)E_{s2}{∥}_{F}^{2}\\ s.t.\; & WY=1_{(N_{c}-1)N}, \end{array}$$

where $P=I_{N(N-1)}-E_{s1}{\left(E_{s1}^{H}E_{s1}\right)}^{-1}E_{s1}^{H}$.

To simplify the objective function in (A2), the following properties of matrix trace are applied [42,43], i.e., ${∥M∥}_{F}^{2}=\mathrm{trace}\left[M^{H}M\right]$, trace[MN]=trace[NM] and $\mathrm{trace}\left[M\Gamma \left(d\right)N\Gamma \left(d\right)\right]=d^{H}\left(M^{T}\circ N\right)d$ for matrices M, N and vectors d with proper dimensions used. The objective function in (A2) can be reformulated as

(A3) $$\begin{array}{cc} ∥P\Gamma \left(\overset{˘}{\gamma }\right)E_{s2}{∥}_{2}^{2} & =\mathrm{trace}\left[{\left(P\Gamma \left(Y\right)E_{s2}\right)}^{H}\left(P\Gamma \left(Y\right)E_{s2}\right)\right]\\ & =Y^{H}\left({\left(E_{s2}E_{s2}^{H}\right)}^{T}\circ P\right)Y. \end{array}$$

Note the fact that P is a projection matrix, the equality $P^{H}P=P$ is also used in the above formula simplification.

Hence, the optimization problem (14) can be simplified as

(A4) $$\begin{array}{cc} \min_{Y}\; & Y^{H}\left({\left(E_{s2}E_{s2}^{H}\right)}^{T}\circ P\right)Y\\ s.t.\; & WY=1_{(N_{c}-1)N}. \end{array}$$

Thus, the derivation is achieved.

## Appendix B

In this Appendix, we provide the detailed derivation for the solution of the optimization problem (15).

We use the Lagrange multipliers method to solve the optimization problem (15). The Lagrangian is defined as

(A5) $$L\left(Y,\lambda \right)=Y^{H}QY+\lambda^{H}\left(WY-1_{(N_{c}-1)N}\right),$$

where $Q={\left(E_{s2}E_{s2}^{H}\right)}^{T}\circ P$ and λ is the Lagrange multiplier with dimension $(N_{c}-1)N\times 1$. Let the partial derivative of (A5) with respect to Y be zero, i.e.,

(A6) $$\frac{\partial L}{\partial Y}=2QY+W^{T}\lambda =0_{N(N-1)}.$$

We have $Y=-\frac{1}{2}Q^{-1}W^{T}\lambda$. Note that $WY=1_{(N_{c}-1)N}$, λ can be expressed as

(A7) $$\lambda =-2{\left(WQ^{-1}W^{T}\right)}^{-1}1_{(N_{c}-1)N}.$$

Then, the solution of the optimization problem in (15) can be given as

(A8) $$\hat{Y}=Q^{-1}W^{T}{\left(WQ^{-1}W^{T}\right)}^{-1}1_{(N_{c}-1)N}.$$
